# Kidney Cancer Diagnosis and Surgery Selection by Machine Learning from CT Scans Combined with Clinical Metadata

**DOI:** 10.3390/cancers15123189

**Published:** 2023-06-14

**Authors:** Sakib Mahmud, Tariq O. Abbas, Adam Mushtak, Johayra Prithula, Muhammad E. H. Chowdhury

**Affiliations:** 1 Department of Electrical Engineering, Qatar University, Doha 2713, Qatar; 2 Urology Division, Surgery Department, Sidra Medicine, Doha 26999, Qatar; 3 Department of Surgery, Weill Cornell Medicine-Qatar, Doha 24811, Qatar; 4 College of Medicine, Qatar University, Doha 2713, Qatar; 5 Clinical Imaging Department, Hamad Medical Corporation, Doha 3050, Qatar; 6 Department of Electrical and Electronics Engineering, University of Dhaka, Dhaka 1000, Bangladesh

**Keywords:** kidney, malignant tumor, cancer surgery, partial nephrectomy, radical nephrectomy, computerized tomography (CT), object detection, classification, machine learning

## Abstract

**Simple Summary:**

Diagnosis is the most important step in treating and managing kidney cancer, requiring accurate identification, localization, and classification of tumor regions. The selection of appropriate surgical procedures for malignant cases is further based on tumor volume and relative severity. In recent years, machine-learning-based approaches have been proposed to localize, quantify, and stratify kidney tumors using contrast-enhanced computed tomography (CT) images. However, previous studies have largely neglected the integration of patient metadata with clinical images to better diagnose and guide surgical interventions. In the current study, we developed a combined clinical and image-based approach to classify kidney cancers using a publicly available dataset. We show that the inclusion of clinical features alongside medical images improves the performance of kidney tumor classification. We further used clinical data together with a machine-learning approach to predict the expected surgical procedure employed in individual kidney cancer patients. In addition to cancer stage and tumor volume, some surprisingly common demographic features were revealed to be key determinants of the surgical procedure later selected for nephrectomy.

**Abstract:**

Kidney cancers are one of the most common malignancies worldwide. Accurate diagnosis is a critical step in the management of kidney cancer patients and is influenced by multiple factors including tumor size or volume, cancer types and stages, etc. For malignant tumors, partial or radical surgery of the kidney might be required, but for clinicians, the basis for making this decision is often unclear. Partial nephrectomy could result in patient death due to cancer if kidney removal was necessary, whereas radical nephrectomy in less severe cases could resign patients to lifelong dialysis or need for future transplantation without sufficient cause. Using machine learning to consider clinical data alongside computed tomography images could potentially help resolve some of these surgical ambiguities, by enabling a more robust classification of kidney cancers and selection of optimal surgical approaches. In this study, we used the publicly available KiTS dataset of contrast-enhanced CT images and corresponding patient metadata to differentiate four major classes of kidney cancer: clear cell (ccRCC), chromophobe (chRCC), papillary (pRCC) renal cell carcinoma, and oncocytoma (ONC). We rationalized these data to overcome the high field of view (FoV), extract tumor regions of interest (ROIs), classify patients using deep machine-learning models, and extract/post-process CT image features for combination with clinical data. Regardless of marked data imbalance, our combined approach achieved a high level of performance (85.66% accuracy, 84.18% precision, 85.66% recall, and 84.92% F1-score). When selecting surgical procedures for malignant tumors (RCC), our method proved even more reliable (90.63% accuracy, 90.83% precision, 90.61% recall, and 90.50% F1-score). Using feature ranking, we confirmed that tumor volume and cancer stage are the most relevant clinical features for predicting surgical procedures. Once fully mature, the approach we propose could be used to assist surgeons in performing nephrectomies by guiding the choices of optimal procedures in individual patients with kidney cancer.

## 1. Introduction

Chronic kidney diseases (CKDs) are progressive conditions that confer gradual loss of renal function, ultimately leading to kidney failure [1]. CKDs are often associated with malignancy and have been a major cause of death throughout the 21st century: rates vary between 7–12% across regions [1], accounting for a staggering 800 million cases globally in 2017 alone [2]. Kidney cancer itself is the 14th most common malignancy worldwide (9th among men) with more than 430,000 cases diagnosed worldwide in the year 2020 [3]. Based on several recent studies [4,5,6], CKDs are now recognized as the primary reason for many kidney cancers [7,8], but also vice-versa [6]. Kidney cancers are normally diagnosed and followed up using routine blood tests, urinalysis, imaging techniques, and occasionally biopsy in more complex cases [1]. Changes in kidney volume and/or tumor burden have also been identified as features of other CKDs [9], including autosomal dominant polycystic kidney disease (ADPKD) [10] and renal artery atherosclerosis (RAS) [11], which often lead to end-stage kidney disease (ESKD) [12]. However, the detection of CKDs through laboratory tests such as the estimated glomerular filtration rate (eGFR) [13] or albumin-to-creatinine ratio (ACR) [14] is a lengthy and complex process with often unreliable results. Consequently, artificial-intelligence (AI)-based systems that can accurately localize, classify, and quantify kidney tumors using clinical images could greatly improve current diagnosis and patient management.

Kidney scans typically comprise X-rays [15], ultrasonography [16], computed tomography (CT) [17], magnetic resonance imaging (MRI) [18], positron emission tomography (PET) [19], and alternative urography techniques [1]. While CT scans are the most common imaging technique for diagnosing kidney cancer [20], each of these methodologies displays clear strengths and weaknesses, leading to specific technical challenges and specialized applications for each. Traditionally, imaging techniques have been used by clinicians to support biomedical testing or as a method of screening complex patients. In recent years, several studies have attempted to use AI-based methods to diagnose kidney diseases using medical images [19,21,22,23,24,25,26,27,28]. Deep-learning techniques have been employed to detect [21,22], quantify [23,24], and classify [25] different types of kidney pathology including tumors [25], stones [24], glomeruli [23], and hydronephrosis [24], based on analysis of CT [25], Ultrasound (US) [24], MRI [26], and other types of clinical image [19,27,28]. 

Kidney cancer diagnosis using abdominal CT images is one of the most crucial tasks in clinical management [19,20,21,22,23,24,25,26,27,28,29,30] but this is often performed in isolation of corresponding patient metadata that is likely relevant to tumor subtype and stage [7,31]. Accurately detecting and classifying tumor regions into subclasses, especially malignant versus benign groups, could therefore be improved by integrating clinical metadata into CKD diagnostics. In addition, doctors often face difficulties in selecting operation type (open, robotic, or laparoscopic) [32], optimal procedure (partial or radical nephrectomy) [33], and surgical approach (transperitoneal or retroperitoneal) [34]. Aiding doctors in selecting the most appropriate surgical intervention based on patient demographic data and other preoperative information would represent a major advance for AI-based tools in this domain. We, therefore, sought to develop a smart system able to reliably classify kidney cancers into major subtypes (especially renal cell carcinoma (RCC)), and guide real-time decision-making regarding surgical approach with the ultimate aim of supporting both clinicians and patients to achieve rapid and robust management from the moment of diagnosis.

In this study, we propose a machine-learning-based approach that combines contrast-enhanced CT images with corresponding clinical metadata from individual patients to classify kidney tumors into major subclasses and guide the selection of an optimal surgical procedure. The main contributions of this study are as follows:

- Computed tomography (CT) images and clinical metadata from the KiTS21 dataset were used to differentiate four major classes of renal cancer: clear cell (ccRCC), chromophobe (chRCC), papillary (pRCC) renal cell carcinoma, and oncocytoma (ONC);
- Tumor subclass predictions were integrated with clinical metadata to determine the optimal surgical approach in malignant cases (radical versus partial nephrectomy);
- To the best of our knowledge, this is the first study to determine kidney tumor subclass using a combination of CT images and corresponding clinical features;
- This pioneering study paves the way for future refinement of tools that can guide surgical interventions in kidney cancer by applying machine-learning algorithms trained on relevant clinical data.

In Section 2, we present a comprehensive review of previous studies dealing with kidney and tumor region of interest (ROI) extraction, segmentation, and classification. We also appraise the current literature on automated classification/surgical decision-making in kidney cancer as well as the associated technical challenges. In Section 3, we describe in detail the various methodologies used by the current study. Section 4 presents the quantitative/qualitative experimental outcomes, as well as supporting visualizations demonstrating the performance of the proposed method. Finally, Section 5 concludes the article and outlines future research directions in this domain.

## 2. Related Work

According to the American Cancer Society (ACS), kidney tumors rank among the ten most common types of malignancy [35]. In recent years, several studies have sought to diagnose renal tumors using contrast-enhanced abdominal CT images. Early attempts processed CT images using manually defined methods, training classifiers to isolate the kidney, identify tumor ROIs, and diagnose/classify cancer type [36,37,38,39]. Other methods have used deep-learning-based techniques to segment kidneys and tumors from CT images [40,41] or extract features from whole CT images, which are limited by noisy background signals that impact diagnostic performance [42,43,44].

The Kidney and Kidney Tumor Segmentation Challenge (KiTS) was first launched in 2019 [45], continued under the subsequent KiTS21 initiative [46], and has now entered a third iteration under KiTS23 [47]. Over this period, challenge participants have proposed various strategies to identify/segment kidney and tumor regions using contrast-enhanced CT images. The top-five solutions from the KiTS19 grand challenge included manual, semiautomated, and fully automated AI-based techniques to extract and segment an ROI from whole CT volumes (rather than processing the entire image), thereby achieving better diagnostic performance [48]. While the main aim of the KiTS challenges was to segment kidney and tumor volumes from 3D-CT scans [48], many subsequent studies have used KiTS or alternative datasets to generate 2D-CT slices for segmentation and/or deep learning-based detection of renal tumors. Yan et al. [49] proposed 3D-MS-RFCNN, a method of segmenting kidney and tumor volumes using the KiTS19 dataset and evaluated performance against an inhouse dataset collected by their team. Hsiao et al. [50] instead proposed a 2D convolutional neural network (CNN)-based feature pyramid network (FPN) to segment kidneys and tumors using 2D-CT slices extracted from the KiTS dataset. They also developed an adaptive window-selection method to robustly determine appropriate radiodensity ranges for kidney CT slices [51]. Lin et al. [52] performed an extensive study in which the authors segmented kidney, tumor, and cyst volumes using 3D-CT images collected from 441 patients. The Crossbar-Net tool proposed by Yu et al. [53] used horizontal and vertical patches to segment kidney tumors from 2D-CT slices. More recently, investigators have begun to employ advanced AI-based techniques such as graph neural networks (GNNs) [54] and generative adversarial networks (GANs) [55] to process CT images and more accurately segment tumors across diverse datasets. However, very few studies in this domain have proposed end-to-end pipelines for kidney cancer diagnosis, which requires accurate classification and grading of the tumors [56].

Accurate identification of tumor subtype and progression/severity are crucial aspects of kidney cancer diagnosis and key determinants of whether surgery will be required [31]. In addition, the precise grading of malignant tumors critically informs the selection of either partial or radical surgery [57,58]. Alzu’bi et al. [59] previously classified normal and tumorous kidneys using 2D-CT slices, then further distinguished between malignant and benign cases within the cancer cohort. Likewise, Zabihollahy et al. [44] differentiated renal cell carcinoma (RCC) from benign tumors (Oncocytoma (ONC) and Angiomyolipoma (AML)) based on manually annotated regions. Han et al. [60] used a similar approach to classify subtypes of RCC, seeking to distinguish clear cell (ccRCC), from chromophobe (chRCC), and papillary cases (pRCC). Kong et al. conducted an extensive study in which they developed the BKC-Net framework [56], which segments renal tumors from kidney CT images and classifies disease into five distinct subclasses. In this study, the authors were also able to differentiate the most commonly occurring renal cell carcinoma (ccRCC) into benign and malignant classes based on the Fuhrman grade [61]. Uhm et al. [25] also proposed a framework that used 3D-CT slices to sort renal tumors into five distinct classes (ccRCC, chRCC, pRCC, ONC, and AML). However, based on our extensive literature review, none of these studies used clinical metadata either alone or in combination with CT image features to classify tumor subtypes.

Kidney cancers—especially malignant cases—often require surgery to remove the tumorous regions. Depending on case complexity, surgeons might elect to perform open, robotic, or laparoscopic surgery [32], using either a transperitoneal or retroperitoneal approach depending on various patient parameters [34,62]. A key decision is whether to opt for partial or radical nephrectomy [33,63], with several previous studies attempting to determine the optimal procedure for various case types. The outcomes of these studies have been decidedly varied, and the choice of the ‘gold standard’ surgical technique for renal cancer remains unclear. Kunath et al. [33] surveyed multiple studies and concluded that partial nephrectomy was associated with decreased patient-survival time, despite not differing from radical nephrectomy concerning surgery-related mortality, cancer-specific survival, and time to recurrence. Conversely, many urologists and nephrologists [63,64] typically opt for partial nephrectomy except in complex cases where total removal of the kidney is necessary, e.g., where cancer has spread outside the renal hilum boundary (or in elderly patients for whom the remaining kidney is likely to provide a sufficient renal function for remaining lifespan) [64]. A major concern among nephrologists regarding radical surgery in mild cases is the risk of future cancers in the remaining kidney, meaning that the affected patient may eventually require dialysis or an organ transplant. In the current study, we investigated whether artificial intelligence (AI) can be used to assist doctors in determining the suitable surgical procedure for patients with renal cell carcinoma (malignant tumors) using a combination of demographic and preoperative clinical data.

## 3. Methodology

**Figure 1:**
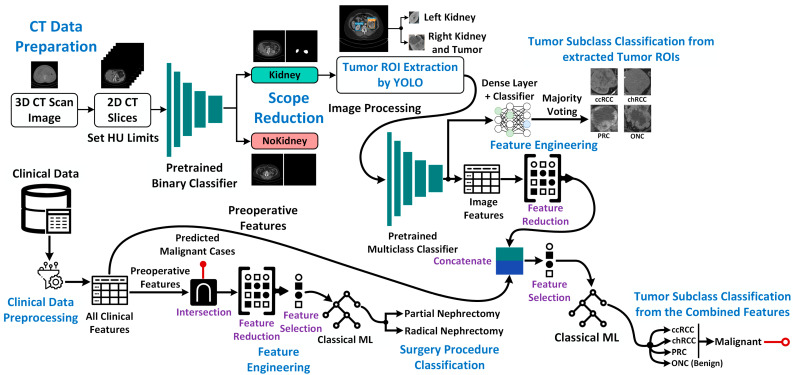
Schematic of the proposed framework for kidney cancer subtype classification using a combination of computed tomography (CT) images and clinical metadata (pipeline begins on the left and extends to the right-hand side of the figure). Surgical procedure classification for malignant renal cell carcinoma (RCC) tumors is a key output from this framework. Major methodological steps are highlighted in blue text.

The overall workflow of this study is presented in Figure 1. In brief, we discuss the KiTS21 dataset, preprocessing steps, scope reduction (removal of non-kidney 2D-CT slices), extraction of regions of interest/ROIs (i.e., kidneys, tumors, and cysts) from CT slices, and tumor classification based on the extracted ROIs (with or without metadata integration). We also discuss the process of determining the optimal surgical procedure for malignant RCC cases via binary classification based on clinical data. The various classical and deep machine-learning tools used in this study have been explained in detail in the respective sections. Finally, we explain the quantitative metrics and qualitative evaluation approaches applied to each experiment.

### 3.1. Dataset Description

In this study, we used the publicly available “Kidney and Kidney Tumor Segmentation Challenge 2021” (KiTS21) dataset [65]. The contrast-enhanced computed tomography (CT) scan dataset was originally published in 2019 as part of the KiTS19 grand challenge [66]. The KiTS21 dataset includes subjects from M Health Fairview [67] and Cleveland Clinic [68] medical centers who underwent either partial or radical nephrectomy between 2010 and 2018. Each of the 544 original cases was reviewed retrospectively to include only those patients who had also undergone a contrast-enhanced CT scan of the entire kidney(s) and corresponding renal tumors. The final compiled dataset contained 3D-CT slices from n = 300 subjects, each with ground truth masks annotated by a group of experts and trainees [65]. KiTS21 is an extended version of the earlier KiTS19 initiative [65], which provided ground-truth annotations or masks for benign cysts alongside masks for kidneys and tumors. KiTS21 also includes ground-truth masks for 90 test subjects from the original KiTS19 challenge. Baseline patient and tumor characteristics of the KiTS21 dataset are provided in Table 1.

### 3.2. Preprocessing of Computed Tomography (CT) Images

The KiTS21 dataset is provided in Neuroimaging Informatics Technology Initiative (NIFTI) file format [69] and compressed using the Gzip method from the GNU project [70], such that multistage, full-body 3D-CT images are available for each subject. Original CT images from n = 300 patients are stored in a repository curated by the challenge organizer [71], while annotated ground-truth segmentation masks for the kidney, tumor, and cyst regions are accessed via GitHub [72]. The ground-truth segmentation masks are also provided in a 3D format to match the CT images slice by slice. Annotations use four levels of pixels (0 to 3) to denote kidney (1), tumor (2), cyst (3), and background (0). The KiTS challenge organizers followed three different schemes to combine pixel-wise annotations from multiple experts: in the ‘AND’ scheme only pixels shared by annotations from all labelers were considered for the final masks; in the ‘OR’ scheme pixels present in annotations from any labeler were considered for ground-truth annotation; in the ‘MAJ’ scheme pixels marked by the majority of annotators were used to generate the final masks. For this study, we elected to use masks generated by the MAJ scheme. The 3D-CT images were unzipped and extracted from the NIFTI files into 3D arrays. A total of 64,603 2D slices were then extracted from n = 300 3D-CT images. Next, the Hounsfield Unit (HU) [51] range for each CT slice was determined. Restricting the CT images to a defined HU range assists radiologists (and deep-learning algorithms) by using the variable radiodensity of different organs to focus on a specific target (in this case kidney). Previous studies have also attempted kidney segmentation using fixed HU ranges [41,48,53,73] or adaptive HU range determination [50]. In their official-challenge article, Heller et al. [48] used a fixed HU range of (−200, 500) which was also employed in the current study. To project pixel values within a given HU range, all values exceeding the upper limit (500) were clipped to this cap, while all values below the lower limit (−200) were clipped to this minimum value. In Supplementary Figure S1, a sample CT slice featuring the total HU range and examples of HU-limited slices are shown.

### 3.3. Scope Reduction through Kidney Instance Classification

**Figure 2:**
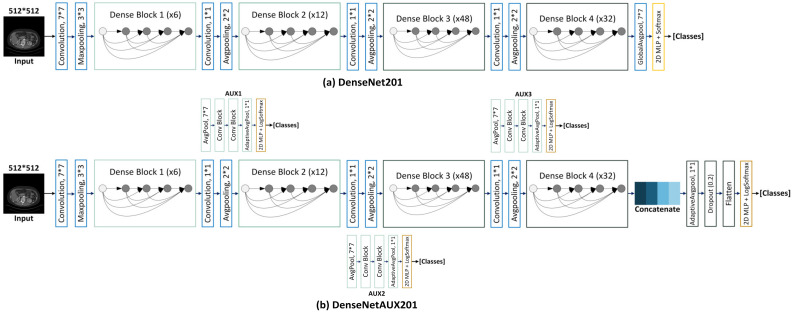
(**a**) The original (**a**) DenseNet201 architecture depicted against the proposed novel (**b**) DenseAUXNet201 having two auxiliary outputs and a modified header. Deeper CNN layers are represented by darker blue shades. MLP layers are shown as yellow blocks.

As proposed by the challenge organizers [48], the ability of deep-learning models to estimate kidney and tumor regions in CT images depends on five main factors: slice thickness, tumor focality, the field of view (FoV), tumor size, and cancer subtype. In this dataset, FoV varies a lot between cases and scanning sites. Some scans contain full-body captures extending from head to toe, thereby introducing a large amount of ‘non-kidney’ information into the dataset. For slices of this type, the ground-truth masks are completely blank, and it becomes hard for the model to determine the kidney and associated tumor or cyst regions. In this study, we dealt with large FoVs using a deep learning-based ‘Scope Reduction’ technique, for which we divided the slices into kidney and non-kidney classes based on ground-truth masks. A deep-learning-based binary classifier was trained to classify the kidney and non-kidney slices (if there was a single nonzero pixel present for a given slice, that was considered a kidney). In a previous study, Cruz et al. [41] performed scope reduction using AlexNet [74], which is one of the earliest CNN-based 2D classification networks. In the current study, we instead tested various state-of-the-art architectures, including ResNet152 [75], DenseNet201 [76], InceptionV3 [77], and MobileNetV2 [78], with pretrained ImageNet weights for scope reduction. This revealed that a modified DenseNet201-based approach with auxiliary losses (DenseAUXNet201, shown in Figure 2) achieved superior performance metrics to the other networks tested.

#### DenseAUXNet201 Architecture

The original DenseNet architecture was proposed in 2018 by Huang et al. [76] as a set of four major variants: DenseNet121, DenseNet169, DenseNet201, and DenseNet264. DenseNet121 and DenseNet201 in particular have been applied to many 2D classification tasks [79,80,81]. DenseNet201 contains 6, 12, 48, and 32 convolutional blocks that are each followed by batch normalization and the ‘ReLU’ activation layer. Transition blocks placed after each denseblock down sample the feature maps to lower dimensions. InceptionV3 is another state-of-the-art model that includes variants containing auxiliary losses that enable monitoring and optimizing the flow of features in intermediate layers. Among pretrained state-of-the-art models used in our study, DenseNet201 performed consistently well across all experiments. To further improve performance, we implemented auxiliary losses with independent-classifier blocks after each of the three intermediate dense blocks (Figure 2) and monitored their outputs for optimization. We also took features from each of the intermediate layers, concatenated these with output from the last layer, and passed the concatenated features to a set of densely connected multilayer-perceptron (MLP) blocks for final classification (all four losses were optimized during training). The proposed DenseAUXNet201 framework improved performance across all evaluation criteria, as discussed in the results section. We also employed ‘*LogSoftmax*’ [82] as the auxiliary and final activation function in DenseAUXNet201 (formulated as shown in Equation (1)).

(1) $$LogSoftmax\left(x_{i}\right)=log\left(\frac{e^{x_{i}}}{\sum_{i}e^{x_{i}}}\right)$$

### 3.4. Region of Interest (ROI) Extraction from 2D-CT Slices

Extracted 2D-CT slices containing kidney footprints can be used directly for tumor subclass classification based on labels, but it is important to note that other organs present in the image heavily impact the decision-making process. To mitigate this, a region of interest (ROI) around the kidney can be extracted from whole CT slices so that multiclass classifiers can focus only on tumorous regions in the next stage. Extracting an ROI for kidney detection or segmentation from CT images has been a common practice across a range of published 2D/3D approaches. For example, three of the four top performers ranked by the KiTS19 challenge extracted ROIs from 3D-CT images, using methods extending from coarse segmentation to extracting volume of interest (VOI) (the 3D equivalent of ROI) [48]. In recent years, different generations of the ‘You Only Look Once’ (YOLO) framework has been used for object detection in many complex scenarios. State-of-the-art YOLO versions (YOLOv5 [83], YOLOv7 [84], etc.) have demonstrated excellent performance in object detection, but are only rarely applied to kidney and/or tumor ROI extraction from CT images. Therefore, in the current study, we tested the ability of YOLOv5 and YOLOv7 frameworks to extract kidney and tumor ROIs from whole 2D-CT slices.

#### 3.4.1. Bounding Box Label Generation from Segmentation Masks

The original KiTS21 annotations feature ground-truth masks for segmentation but do not contain bounding-box labels as required for training YOLO frameworks. We, therefore, used the segmentation masks to generate three classes of rectangular bounding boxes for the YOLO framework, corresponding to the three-pixel levels used to denote kidney, tumor, and cyst regions. First, contours were generated and unified around the masks, then bounding boxes were positioned to encompass the four extreme corners of these contours. During the generation of ground-truth bounding boxes, we observed that tumor regions appeared very distorted or blurry if these were either very irregular in shape or small in size (resulting in upsampling/interpolation to fixed dimensions). To avoid this impacting model performance, the bounding boxes were adjusted to form squares based on the highest dimension of length or width. Next, we removed any slices in which the bounding box area for a kidney region was less than 48 pixels height/width or <1% of the total slice area (512 × 512 = 262,144). The overall bounding box generation process is summarized in Supplementary Figure S2. The YOLO framework accepts four parameters alongside the class label (normalized height/width and *x*/*y* coordinates of the bounding box center point), whereas contours provide height, width, and *x*/*y* coordinates, and define the origin (0, 0) as the left-uppermost corner of the bounding box. We, therefore, generated labels for YOLO using Equations (2)–(5);

(2) $$Normalized\;x-coordinate\;\left(center\right)=\frac{\left(x-coordinate+\frac{box\;width}{2}\right)}{image\;width}$$

(3) $$Normalized\;y-coordinate\;\left(center\right)=\frac{\left(y-coordinate+\frac{box\;height}{2}\right)}{image\;height}$$

(4) $$Normalized\;box\;width=\frac{box\;width}{image\;width}$$

(5) $$Normalized\;box\;height=\frac{box\;height}{image\;height}$$

For the YOLO framework, labels are saved in text files linked to each image (with matching file names). Since we used YOLO models pretrained on the COCO dataset [85], images were resized to the same (640 × 640) format during training.

### 3.5. Kidney Tumor Subtype Classification from Extracted CT ROIs

**Figure 3:**
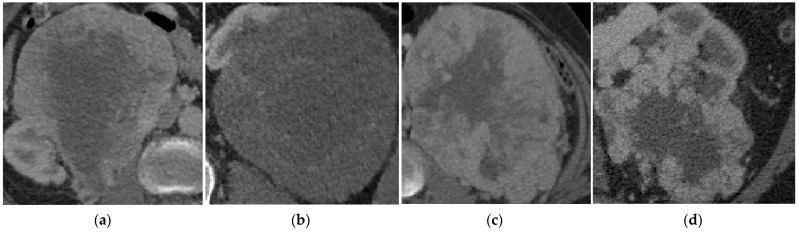
Example kidney tumor subtype visualization. From left, (**a**) clear cell (ccRCC), (**b**) Chromophobe (chRCC), (**c**) Papillary (pRCC) renal cell carcinoma, and (**d**) Oncocytoma (ONC).

The KiTS metadata file available via GitHub [72] contains demographic and clinical information for each of the n = 300 subjects. These cases include 13 different types of kidney tumors (except for n = 1 individual classed as ‘other’). The predominant classes of renal tumors in this dataset are clear cell (ccRCC—204 cases), papillary (pRCC—28 cases), chromophobe (chRCC—27 cases), oncocytoma (ONC—16 cases), and angiomyolipoma (AML—5 cases). Since the total number of image samples from n = 5 AML cases was inadequate for deep-learning-based image classification, these were removed from the dataset. To further reduce class imbalance among the remaining groups, we augmented the training images in each fold using random rotation (−90° to 90°) and random vertical/horizontal flip (Supplementary Table S1). The final task was therefore a four-group classification problem requiring differentiation between ccRCC, pRCC, chRCC, and ONC. A total of n = 3 ccRCC cases were excluded due to extremely small or highly distorted tumor ROIs (as described in Section 3.4.1) leaving a total of n = 272 cases for use in this study. Figure 3 shows one representative tumor sample per class for visualization (ROI extracted and resized).

The prepared dataset was trained and evaluated using a 2D-CNN-based multiclass classifier. The models used for this task were the same as those employed for scope reduction (ResNet152 [75], DenseNet201 [76], InceptionV3 [77], MobileNetV2 [78], and DenseAUXNet201 with pretrained ImageNet weights). For this application, the last linear layer before final activation included four output neurons to match the number of classes. For subject-wise evaluation, ground truth and predicted image labels were transformed into subject labels through majority voting.

### 3.6. Tumor Subtype and Surgery Procedure Classification from Clinical or Combined Data

Clinical metadata in the KiTS dataset is provided in JavaScript object notation (JSON) format which was converted into a tabular format for preprocessing. The clinical features can be divided broadly into preoperative, intraoperative, and postoperative categories (with preoperative features also containing demographic information). For this task, the primary aim is to boost the tumor subtype classification performance of machine-learning algorithms by combining clinical data with features extracted from the CT images. Subsequently, these clinical features were also used to classify whether radical or partial nephrectomy was selected when operating on malignant tumors (RCCs).

#### 3.6.1. Clinical Data Pre-Processing

After extracting clinical metadata, we used only preoperative elements to inform our diagnostic framework, since only presurgical features would be available to guide decision-making in real-life scenarios. In addition, intraoperative characteristics and postoperative outcomes arise from surgical decisions taken by a diverse range of clinicians, rather than being objective features of individual patients and kidney tumors. Key preoperative clinical parameters included tumor size (radiographic and pathologic), demographic information (gender, age, and body mass index (BMI), as well as alcohol/smoking/tobacco history, and the presence of 19 common comorbidities (e.g., myocardial infarction, congestive heart failure, etc.). For predicting surgical procedure, we also included tumor subtype and Fuhrman grade [57] since operation choice critically depends on case complexity (including cancer subclass and severity). Within the tabulated dataset, age and BMI were range-normalized between 0 and 1 to remove bias. The only other numerical features present were radiographic and pathologic measures of tumor volume. Categorical features were transformed into numerical values by replacement. KiTS21 metadata does not provide Fuhrman tumor grade but does specify tumor, node, and metastasis (TNM) cancer stages [86], as proposed by the American Cancer Society (ACS) [35]. We, therefore, transformed these values to achieve the four-stage Fuhrman nuclear grade for kidney cancer severity [57] (see Table 2).

In the TNM grading system, T refers to the size and extent of the primary tumor, N indicates the number of malignant lymph nodes nearby, and M denotes whether cancer has metastasized (i.e., severe cases of tumor spreading to remote organs such as lungs, liver, or brain) [87]. Based on Table 2, if the tumor size has reached T-stage 4, cancers with M = 0 and any N value are classified as Fuhrman stage IV. Alternatively, when there are signs of metastasis (M = 1), cancer will always be considered stage IV regardless of T and N values. Stage III cancers depend on both T and N scores unless tumorous tissues are present in one or more nearby lymph nodes (i.e., default stage III disease). Based on these unification criteria, n = 259 patients with malignant tumors included n = 171 stage I cases, n = 17 stage II, n = 56 stage III, and n = 15 stage IV. The n = 16 ONC cases were marked as benign and n = 3 ccRCC cases with small/distorted tumor ROIs were removed from the dataset (as described in Section 3.4.1). Of the remaining cases, n = 163 underwent partial nephrectomy while n = 93 patients were subjected to radical nephrectomy. A total of 29 raw clinical features were used for tumor subtyping and surgical procedure classification.

#### 3.6.2. Feature Engineering

Image features were extracted from the penultimate layer of the best-performing 2D-CNN-based classifier (DenseAUXNet201). The final classifier was removed for feature extraction, and a total of 4480 features were obtained from each image (using the latent layer of DenseAUXNet201). Features from all images for a test subject were concatenated and reduced through principal component analysis (PCA) [88]. For the clinical data, highly correlated features were removed based on a threshold of 0.85 cross-correlations. Finally, the most influential features were ranked using three distinct techniques [89]; XGBoost [90], random forest [91], and extra trees [92] (in addition to combining these with existing clinical features through horizontal concatenation). For surgical procedure selection, we employed the same feature engineering approach to determine the most relevant features before classification.

#### 3.6.3. Classical Machine-Learning Algorithms

For this study, we used different types of ML-based classifiers for both tumor sub-typing (combined approach) and surgical procedure assignment: logistic regression, k-nearest neighbors (kNN), support vector machine (SVM), random forest, extra trees, linear discriminant analysis (LDA), gradient boost (GradBoost) [93], adaptive boost (AdaBoost), XGBoost [90], light GBM (LGBM) [94], ElasticNet, ridge, and multilayer perceptron (MLP).

### 3.7. Quantitative Evaluation Metrics

We used two broad types of machine-learning applications for classification (deep or standard ML) and object detection (deep ML). Distinct evaluation criteria were applied for each approach.

#### 3.7.1. Classification

The performance of the classifiers (both standard and deep ML) was evaluated based on accuracy, precision, recall/sensitivity, specificity, and F1-score. We initially extracted the number of true positive (*TP*), true negative (*TN*), false positive (*FP*), and false negative (*FN*) labels from the confusion matrix, then these metrics were formulated for subject-wise evaluation based on Equations (6)–(10);

(6) $$Accuracy=\frac{TP+TN}{TP+TN+FP+FN}$$

(7) $$Precision=\frac{TP}{TP+FN}$$

(8) $$Recall\;or\;Sensitivity=\frac{TP}{TP+FP}$$

(9) $$Speicificity=\frac{TN}{TN+FP}$$

(10) $$F1-score\equiv 2*\frac{Precision*Recall}{Precision+Recall}=\frac{2TP}{2TP+FP+FN}$$

All metrics except accuracy were weighted to deal with class imbalance (a common issue with this type of task). For accuracy, we reported the overall macro value calculated from the confusion matrix for the entire dataset. We also show the confusion matrix for the best-performing model at individual stages of each task.

#### 3.7.2. Object Detection

The metrics most commonly used to assess the accuracy of bounding-box generation by YOLO frameworks are precision, recall, and mean average precision (*mAP*), at various intersections over union (IoU) levels. Precision and recall metrics have been defined in Equations (7) and (8), respectively (computed based on ground truth and predicted bounding box coordinates). Average precision (*AP*) can be defined as a technique to summarize the whole precision-recall curve into a single value, as formulated in Equation (11).

(11) $$AP=\overset{k=n-1}{\underset{k=0}{\sum }}\left[Recalls\left(k\right)-Recalls\left(k+1\right)\right]*Precision\left(k\right)$$

*AP* is the weighted sum of precision at each threshold, where weight is the increase in recall. In this case, ‘n’ is the number of thresholds, hence Recall(n) = 0 and Precision(n) = 1. The *mAP* value can be derived from *AP* by calculating the average *AP* from all the classes. If there are ‘c’ number of classes, *mAP* can be defined as:

(12) $$mAP=\frac{\sum_{i=1}^{i=c}AP_{k}}{c}$$

The current study includes three distinct classes, namely kidney, tumor, and cyst regions. In this case, the sum of *AP* in all classes will be divided by three to obtain *mAP*, which is a key metric of object detection performance (i.e., unbiased generation of accurate bounding boxes). Both large/obvious objects and small/challenging cases are captured by *mAP* calculation. For YOLO, the common practice is to measure *mAP* at IoU threshold 0.5 and IoU range 0.5–0.95 [95], which is a robust performance metric for object detection frameworks when considering moderately to highly challenging cases.

### 3.8. Qualitative Evaluation

To further assess YOLO performance, we plotted ground truth and estimated bounding boxes side-by-side for various types of sample cases. We also plotted the performance curves (F1, P, PR, and R) [96] of the best-performing YOLO model. To illustrate the performance of the deep CNN-based image classifiers, we used class activation mapping (CAM) [97] to generate a weighted activation map for each image based on a trained classifier. Current state-of-the-art CAM techniques include GradCAM [98], GradCAM++ [99], Smooth GradCAM++ [100], and ScoreCAM [101]. We employed ScoreCAM which uses the trained model to generate weighted heatmaps of input test images in each class and visualize the classifier learning process (unlike other versions such as GradCAM, which use generic algorithms for this task). This analysis can improve understanding and validation of model performance when considered alongside quantitative metrics.

## 4. Experimental Results

Here we present the quantitative and qualitative outcomes of tumor subclass classification by deep learning from kidney CT images (with or without integration of clinical metadata). Assignment of surgical procedures for malignant tumors based on clinical parameters is also assessed.

### 4.1. Kidney Tumor Classification

As depicted in Figure 1, our proposed method for renal tumor classification from CT images consists of three main steps: scope reduction, kidney/tumor ROI extraction, and tumor subtype classification using deep ML algorithms. In the combined approach, we concatenate both image and clinical features from the same subjects to classify kidney tumors using classical ML techniques. To ensure robust evaluation, we stratified the dataset of n = 300 subjects into five folds in a subject-wise fashion (see Supplementary Table S1). In each fold, the test set contains 60 subjects (20% of the total dataset). The remaining n = 240 cases are then divided into training and validation sets (80:20 ratio). The ranges specified in Supplementary Table S1 were applied in all subsequent experiments.

#### 4.1.1. Scope Reduction through Binary Classification

Scope reduction was performed to remove non-kidney CT slices from the dataset, thus allowing the object detection networks to focus on kidney, tumor, and cyst ROI extraction without any impact of irrelevant slices. We extracted 64,603 2D-CT slices from a total set of 300 CT images. Among these, approximately 41,675 (64.5%) were non-kidney slices, which would represent a major confounder for object detection if not removed. Table 3 shows the combined fivefold performance of the deep-learning-based binary classifiers when processing subject-independent test sets.

The proposed DenseAUXNet201 framework displayed the best performance across all evaluation metrics and achieved the lowest number of missed cases. While only a modest increase in performance was noted, this nonetheless represented a significant improvement in the detection and removal of non-kidney slices (and vice-versa).

**Figure 4:**
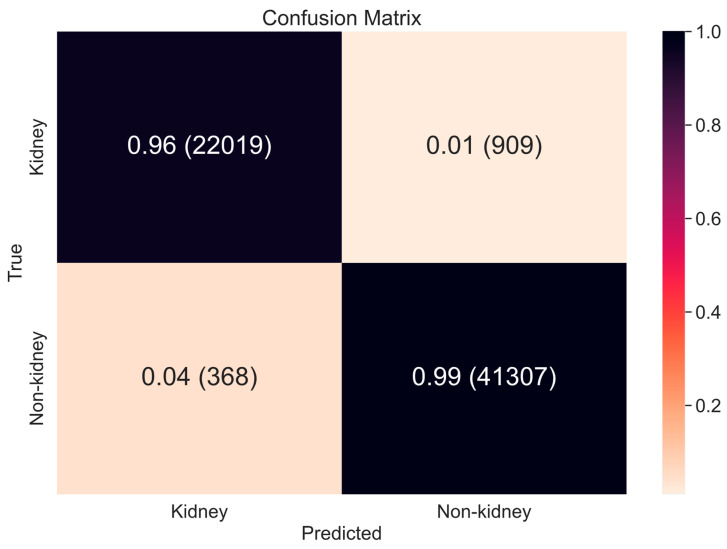
Confusion Matrix of scope reduction by DenseAUXNet201 binary classifier.

As plotted in Figure 4, we observed that 909 slices were misclassified as ‘non-kidney’ and 368 slices were misclassified as ‘kidney’, amounting to just 1277 missed cases among 64,603 total slices. Based on our review of experimental outcomes, most missed cases were extreme, i.e., kidney regions were very small, or the model detected other organs as trace kidney signals.

**Figure 5:**
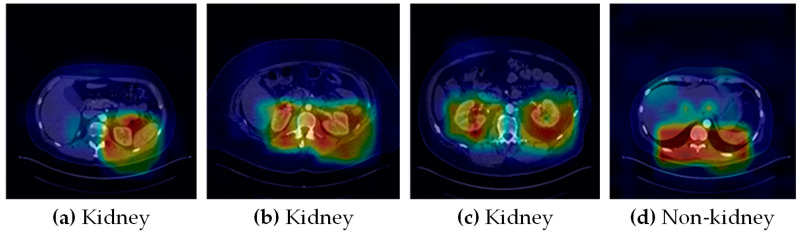
ScoreCAM-generated heatmaps of the (**a**–**c**) kidney and (**d**) non-kidney slices.

In Figure 5, the learning capabilities of the trained DenseAUXNet201 model are illustrated using ScoreCAM-generated weighted heatmaps from the final layer (before the last classifier block) as applied to representative regions or features. For slices with one or two kidneys, the model is focusing on the relevant regions, whereas for slices lacking kidneys, the model is learning to classify based on alternative tissues and features (e.g., lungs).

#### 4.1.2. Region of Interest (ROI) Extraction from 2D-CT Slices using YOLO

We experimented with six models from two different generations of YOLO to extract kidney, tumor, and cyst ROIs using the scope-reduced 2D-CT dataset (YOLOv5 [83] variants YOLOv5s, YOLOv5m, YOLOv5l and YOLOv5x, plus YOLOv7 [84] variants YOLOv7 and YOLOv7x). Here, ‘s’, ‘m’, ‘l’, and ‘x’ denote small, medium, large, and extra-large model variants, respectively. We tested YOLO networks on the fivefold split dataset as described in Supplementary Table S1 and the results are provided in Table 4. While older YOLOv5 models achieved better precision than YOLOv7 variants, the more recent models displayed superior *mAP* at the IoU threshold of 0.5 and range of 0.5–0.95 [95]. Lower precision leads to more false positives from YOLOv7 models compared with YOLOv5 variants, but the higher *mAP* values indicate more efficient detection of very small and/or challenging regions (crucial in the case of tumor identification). We, therefore, selected basic YOLOv7 as the best-performing model for our experiments.

**Figure 6:**
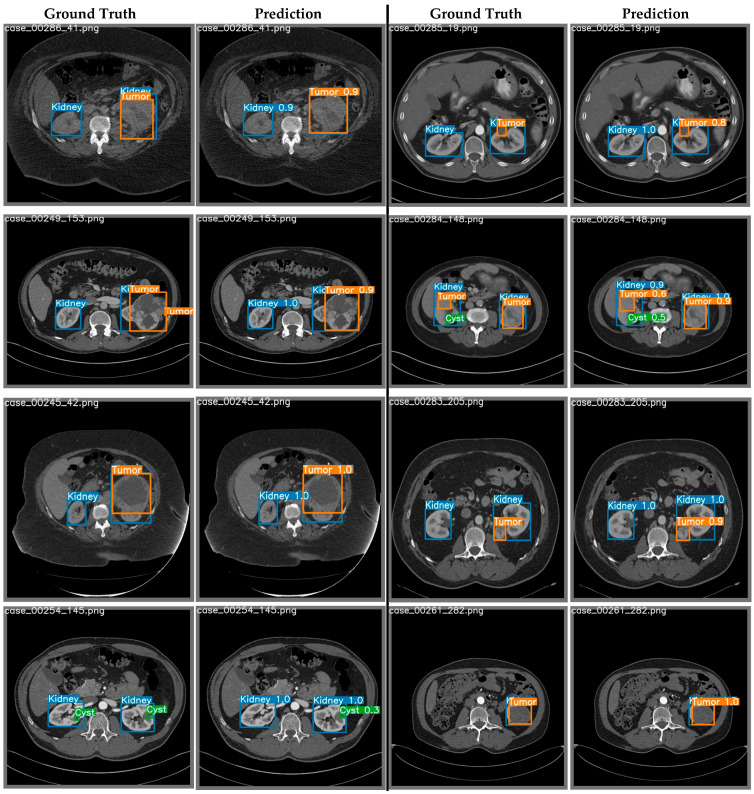
Example ground-truth labels vs. prediction plots from the best performing YOLOv7 model. Shown is the detection of kidney, tumor, and cyst ROIs from CT slices (displayed for qualitative evaluation).

While YOLO worked indirectly as a classifier for the identification of tumors and cysts, the cyst detection performance was very low due to the tiny size of these structures (and the limited number of examples in the training/test datasets). This was not the case for the processing of tumor ROIs i.e., the primary goal of this study. Example outputs from the YOLOv7 network alongside their corresponding ground-truth values are shown in Figure 6. The performance curves of the YOLOv7 model (e.g., F1, precision-confidence, precision-recall, and recall-confidence) are plotted in Supplementary Figure S3. The plots display curves for each of the three classes as well as the combined outcome.

#### 4.1.3. Kidney Cancer Subtype Classification from Tumor ROIs and Clinical Metadata

We next compared the performance of kidney cancer classification using extracted tumor ROIs (image-based) and the combined approach (image + clinical features). After curation, the dataset contained n = 272 cases for classification. Data were split subject-wise in the same way for all experiments (Supplementary Table S1) so that features from both pipelines could be directly compared. We resized the ROIs and augmented training sets in each fold to minimize class imbalance (due to the relative abundance of real-world ccRCC samples). Here, the model remains biased towards the ccRCC if the class imbalance is not mitigated through proper augmentation. The image-wise outcomes were converted into subject-wise results through majority voting on the outcomes for each patient.

**Figure 7:**
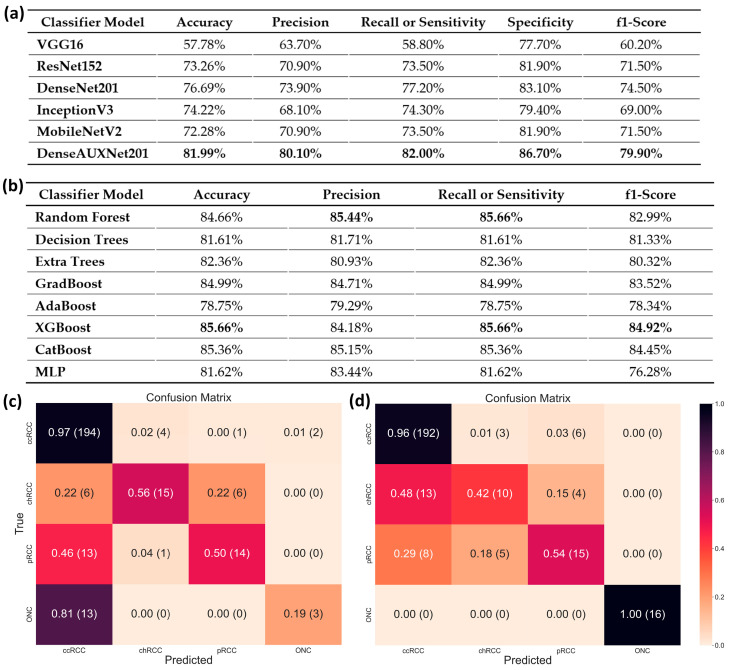
Subject-wise renal tumor subtype classification outcomes. Classification evaluation metrics for (**a**) CT-based classification using deep ML (the best performance for each metric has been made bold), and (**b**) combined features approach using classical ML. Confusion matrix for (**c**) CT-based classification, and (**d**) combined features approach.

**Figure 8:**
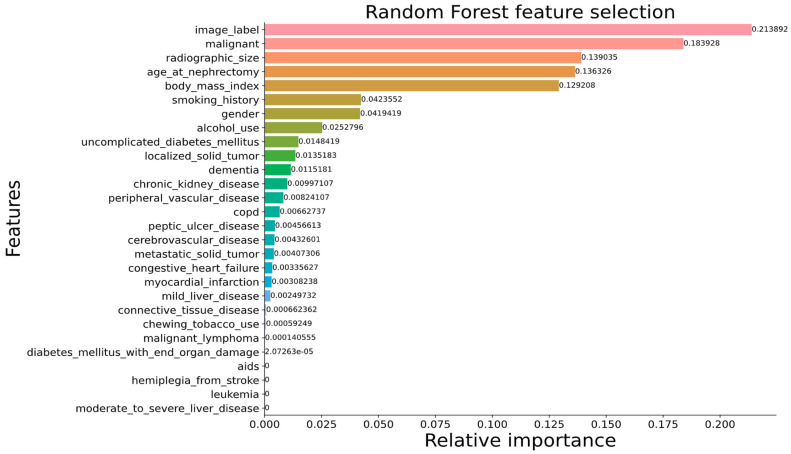
Key determinants of kidney tumor classification through the combined approach as identified by random forest-based feature ranking. Here, more reddish bars represent higher magnitudes while more greenish bars represent lower magnitudes.

Figure 7a,c show that our proposed DenseAUXNet201 model achieved better performance than the current state-of-the-art classification models across all evaluation criteria. The custom auxiliary losses and feature concatenation from intermediate layers boosted its performance significantly. Next, we proceeded to combine the clinical and engineered image features through concatenation. A total of 4480 features (based on the latent dimension of DenseAUXNet201) were extracted from the tumor ROIs, which were efficiently compressed through PCA to only 20 features and combined with the 29 raw clinical features. The pipeline automatically removed ‘pathologic size’ due to high concordance with the ‘radiographic size’ feature. The remaining 48 features were ranked using the three feature-ranking algorithms described in Section 3.6.2, and only the top 20 features were found to be adequate for the optimum performance, as shown in Figure 8, based on the random forest-based feature selection technique. The outcomes from the XGBoost and the extra trees feature ranking techniques are provided in Supplementary Figure S4. Based on the horizontal bar chart in Figure 8, the tumor class from the images was the most prominent feature affecting the classifier, followed by the tumor malignancy marker stored in the clinical data. Tumor size, demographic features such as age, BMI, gender, and habitual features such as smoking or drinking history were also marked as important factors behind tumor classes by the feature-ranking algorithm. Next, a set of classical ML networks were used to classify cases based on these 20 top-ranked features, the results of which are provided in Figure 7b,d.

These analyses revealed that random forest, XGBoost, and CatBoost algorithms performed far better than the other methods. We selected XGBoost due to its higher F1-score (equivalent to the Sørensen dice coefficient used for segmentation or object detection tasks [102]). From the confusion matrices plotted in Figure 7a,b, we observed that combining image features with clinical ones significantly improved in separating the ONC class due to its nonmalignant or benign nature. In contrast, pRCC cases were classified more effectively after feature combination, ccRCC classification performance remained unchanged overall while chRCC identification notably worsened (confused with ccRCC). This occurred primarily due to the class imbalance in the dataset, which could not be fully mitigated through augmentation.

### 4.2. Surgical Procedure Determination from Clinical Data for Malignant Tumors

**Figure 9:**
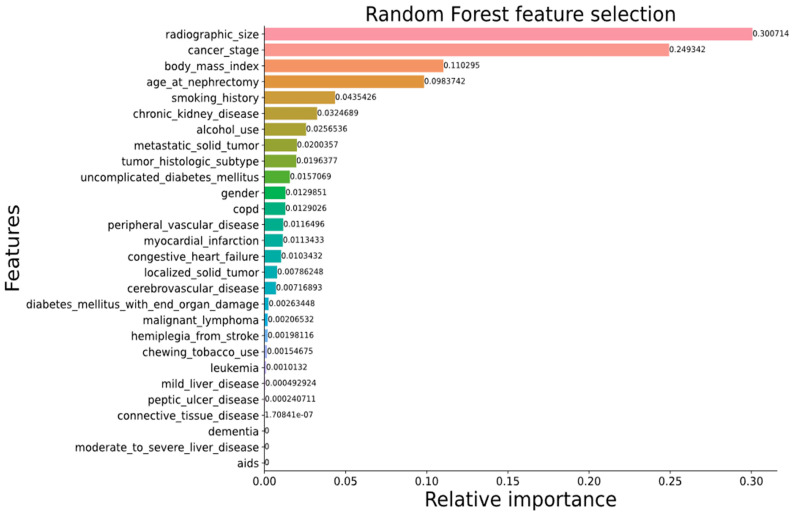
Key determinants of surgical procedure selection as identified by random forest-based feature ranking. Here, more reddish bars represent higher magnitudes while more greenish bars represent lower magnitudes.

As discussed earlier, the raw clinical features included 29 parameters with the potential to influence the classification of kidney tumor surgical procedures. First, we removed highly correlated features to avoid redundancy and reduce overfitting. As before, the ‘pathologic size’ feature was detected as being highly correlated with ‘radiographic size’ and was therefore removed. The remaining 28 features were then ranked based on their relevance to surgical procedure selection. Among the three feature ranking techniques experimented with in this study (XGBoost, random forest, and extra trees), we opted to use random forest (Figure 9) due to it providing more relevance to practical scenarios.

Feature ranking by random forest (Figure 9) and extra trees (Supplementary Figure S5b) generated similar outputs for both cases (tumor and surgery classification) due to both being tree-based techniques. The most important features ranked by random forest were radiographic size/tumor volume and Fuhrman stage, followed by patient BMI, age, smoking habits, history of CKDs, etc. When using XGBoost (Supplementary Figure S5a), importance was also assigned to the presence of a solid metastatic tumor and additional features such as chronic obstructive pulmonary disease (COPD), which are often linked with kidney pathology [103,104]. Practically, surgeons take a more radical approach for complicated end-stage cancers and large tumors [33,63]. Clinicians also prefer a more radical approach for elderly patients with high BMIs [64]. Long-term smoking habits have been linked with kidney cancers in recent studies [105,106], especially renal cell carcinoma (RCC) [106]. On the other hand, alcohol consumption was found to be a vital cause of ESKDs, especially RCCs [107,108]. So, the top-ranked features from the feature ranking algorithm match closely with relevant literature, which validates our classifier’s performance.

**Figure 10:**
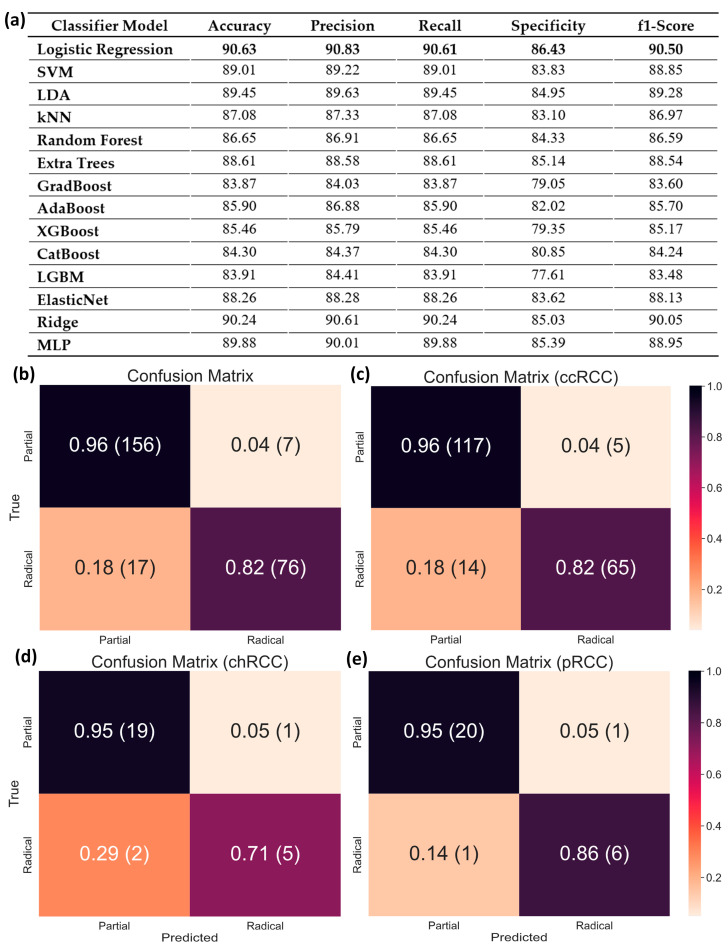
Subject-wise classification results for surgical procedure selection in malignant cases (partial or radical nephrectomy decision based on clinical data). (**a**) Performance evaluation metrics for all classical ML-based classifiers, confusion matrix for (the best performance for each metric have been made bold) (**b**) all 256 malignant cases, (**c**) ccRCC cases, (**d**) chRCC cases, and © pRCC cases.

We finally chose the top 20 features as ranked by random forest for the surgery classification task. We removed any feature with <0.1% relevance to the target and then trained the classifiers over 1000 epochs or until convergence (then repeated this process for all five folds). Outcomes were evaluated in terms of overall accuracy and weighted precision, recall/sensitivity, specificity, and F1-score. The combined (averaged) fivefold classification results are provided in Figure 10. Among the 14 classifiers used in this study, logistic regression, linear discriminant analysis (LDA), support vector machines (SVM), ridge, and multilayer perceptron (MLP) performed far better. We selected traditional logistic regression for our study due to this being a simple classifier with high performance for the current application. In Figure 10b–e, we show the overall confusion matrix for surgical procedure classification across the entire dataset, as well as individually for ccRCC, chRCC, and pRCC cases. We observed that the tumor subtype did not bias the performance of the classifiers in selecting the appropriate surgical procedures. Just 7 partial cases were misclassified as radical, whereas 17 radical cases were misclassified as partial. Further refinement of this AI tool can therefore aid clinicians in making prompt surgical decisions based on preoperative clinical data.

According to Cancer Research UK [109], surgeons take objective decisions on the optimal surgical approach for kidney cancers primarily based on parameters such as patient health and fitness, size and location of the tumor, cancer stage, presence of lymph nodes, and metastasis. While these parameters from the KiTS21 metadata have been included as features in our classifier, there can be other uncommon but important factors influencing the surgical procedure. For example, the American Cancer Society (ACS) discusses surgical approaches for recurrent cancers [110]. These factors are out of the scope of this study since the KiTS21 metadata does not contain this information at all or is not adequate for the classifier to make robust decisions. Solving such problems requires collecting a custom, objective-driven dataset from a good number of kidney cancer surgery patients. Nevertheless, with more dedication, our proposed approach has the potential to be an important AI companion to cancer surgeons.

## 5. Conclusions

AI-based tumor detection and subtype classification can help guide medical and surgical decision-making for kidney cancer patients. Locating kidneys, tumors, and cysts from CT slices using deep-learning approaches is itself a useful application for aiding or training clinicians and radiologists during a cancer diagnosis. Provided acceptable levels of performance, AI-based smart systems can inform the choice of therapy or surgical procedure in kidney cancer, thereby preserving renal function in patients who require only partial nephrectomy or saving the lives of patients with end-stage cancers requiring radical intervention. The primary limitation of this study was the generality of the KiTS dataset. The proposed AI tool could be significantly improved by collecting a large, objective-driven dataset designed to refine this approach. The collected custom dataset should be more balanced in terms of tumor subtypes and cancer stages, ideally containing thousands of diverse cases to make the AI tool more robust for a large population group. More clinical biomarkers, patient medical history, immediate relevant parameters to the surgical table, and other important habits and comorbidities should also be recorded to render this tool more reliable and further improve performance.

## Figures and Tables

**Table 1:** Baseline patient and tumor characteristics of the KiTS21 dataset.

Attribute	Values (N = 300)
Demographic	
Age (years)	60 (51, 68)
BMI ($\mathrm{kg}/m^{2}$)	29.82 (26.16, 35.28)
Gender (M/F)	(180/120) ≡ (60%/40%)
Tumor Size	
Tumor Diameter ^1^ (cm)	4.2 (2.600, 6.125)
Tumor Volume ^1^ (${\mathrm{cm}}^{3}$)	34.93 (9.587, 109.7)
Subtype	
Clear Cell RCC (ccRCC ^2^)	203 (67.7%)
Papillary RCC (pRCC)	28 (9.3%)
Chromophobe RCC (chRCC)	27 (9%)
Oncocytoma (ONC)	16 (5.3%)
Angiomyolipoma (AML)	5 (1.7%)
Other	21 (7%)
Malignancy	
Benign	25 (8.3%)
Malignant	275 (91.7%)
Pathological T-stage	
0	25 (8.3%)
1 (1a, 1b)	180 (60%)
2 (2a, 2b)	20 (6.7%)
3	70 (23.3%)
4	5 (1.7%)
Pathological N-stage	
0	122 (40.7%)
1	11 (3.7%)
2	1 (0.3%)
X ^3^	166 (55.3%)
Pathological M-stage	
0	130 (43.3%)
1	32 (10.7%)
X ^3^	138 (46%)
Renal Anatomy	
Normal	293 (97.7%)
Solitary	5 (1.67%)
Horseshoe	2 (0.67%)
Surgery Type	
Open	79 (26.3%)
Robotic	172 (57.3%)
Laparoscopic	49 (16.3%)
Surgical Procedure	
Radical Nephrectomy	112 (37.3%)
Partial Nephrectomy	188 (62.6%)
Surgical Approach	
Transperitoneal	244 (81.3%)
Retroperitoneal	56 (18.7%)

^1^ In cases of multiple tumor instances, only the largest measured tumor has been reported. ^2^ RCC: Renal Cell Carcinoma. ^3^ X: Unknown or unsure.

**Table 2:** Fuhrman cancer stage determination using the TNM grading system.

Cancer Stage	T	N	M
I	1	0	0
II	2	0	0
III	3	0	0
	1–3	1–3	0
IV	4	Any	0
	Any	Any	1

**Table 3:** Scope reduction performance of deep-CNN-based binary classifiers. The best performance for each metric has been made bold.

Models	Performance					Missed Cases
	Accuracy	Precision	Recall	Specificity	F1-Score	
ResNet152	97.53%	97.55%	97.54%	96.28%	97.54%	1592
DenseNet201	97.95%	97.95%	97.95%	97.07%	97.95%	1325
InceptionV3	97.75%	97.75%	97.75%	96.66%	97.75%	1457
MobileNetV2	97.64%	97.65%	97.64%	96.46%	97.65%	1524
DenseAUXNet201	**98.02%**	**98.03%**	**98.02%**	**97.13%**	**98.03%**	**1277**

**Table 4:** YOLO performance during kidney, tumor, and cyst ROI extraction from 2D-CT slices. The best performance for each metric has been made bold.

Model	Class	Precision	Recall	*mAP* at 0.5	*mAP* at [0.5:0.95]	Inference Time (ms)	Number of Parameters (Millions)	Layers	GIGA-FLOPS
YOLOv5s	Kidney	0.982	0.965	0.986	0.920	14	7.02	157	15.8
YOLOv5m		**0.983**	0.968	0.987	0.922	17.3	20.86	212	47.9
YOLOv5l		**0.983**	0.967	0.987	0.923	31.2	46.12	267	107.7
YOLOv5x		0.981	0.969	0.987	**0.924**	20.9	86.2	322	203.8
YOLOv7		0.975	0.974	**0.988**	0.918	13.9	36.50	314	103.2
YOLOv7x		0.965	**0.975**	**0.988**	0.917	**13.3**	70.80	362	188.0
YOLOv5s	Tumor	0.755	0.639	0.704	0.428	14	7.02	157	15.8
YOLOv5m		0.763	0.656	0.712	0.450	17.3	20.86	212	47.9
YOLOv5l		**0.798**	0.664	0.726	0.476	31.2	46.12	267	107.7
YOLOv5x		0.758	0.655	0.713	0.470	20.9	86.2	322	203.8
YOLOv7		0.773	0.718	**0.756**	**0.525**	13.9	36.50	314	103.2
YOLOv7x		0.761	**0.719**	0.755	0.510	**13.3**	70.80	362	188.0
YOLOv5s	Cyst	0.44	0.184	0.183	0.113	14	7.02	157	15.8
YOLOv5m		**0.513**	0.214	0.182	0.113	17.3	20.86	212	47.9
YOLOv5l		0.483	0.181	0.192	0.120	31.2	46.12	267	107.7
YOLOv5x		0.482	0.225	0.222	0.137	20.9	86.2	322	203.8
YOLOv7		0.414	0.34	**0.278**	**0.182**	13.9	36.50	314	103.2
YOLOv7x		0.405	**0.36**	0.277	0.179	**13.3**	70.80	362	188.0

## Data Availability

The data used in this study can be made available upon a reasonable request to the corresponding author.

## Supplementary Materials

The following supporting information can be downloaded at: https://www.mdpi.com/article/10.3390/cancers15123189/s1; Figure S1. 2D Kidney CT slices of varying HU. (a) Total HU range, original slice, (b) range between [−200, 500], as used in the current study, and (c) [−30, 200]; Figure S2. Bounding box creation process for training YOLO frameworks; Figure S3. YOLOv7 model performance curves plotted for each object type (kidney, tumor, and cyst), alongside combined performance; Figure S4. XGBoost (a) and Extra Trees (b) classifier-based feature selection outcomes for the kidney tumor classification. Ranked features from the Extra trees and Random Forest algorithms were very similar; Figure S5. XGBoost (a) and Extra Trees (b) classifier-based feature selection outcomes for the surgery procedure classification. Ranked features from the Extra trees and Random Forest algorithms were very similar; Table S1. Subject-wise split five-fold information regarding the subject range.
