# Supplementary material for: Kidney Cancer Diagnosis and Surgery Selection by Machine Learning from CT Scans Combined with Clinical Metadata
Source: Cancers (Basel). 2023 Jun 14;15(12):3189. doi: 10.3390/cancers15123189 (PMC10296307; doi:10.3390/cancers15123189)
Supplement: Supplementary file 1 [file cancers-15-03189-s001.zip › cancers-2403359-supplementary.pdf]

Supplementary Materials

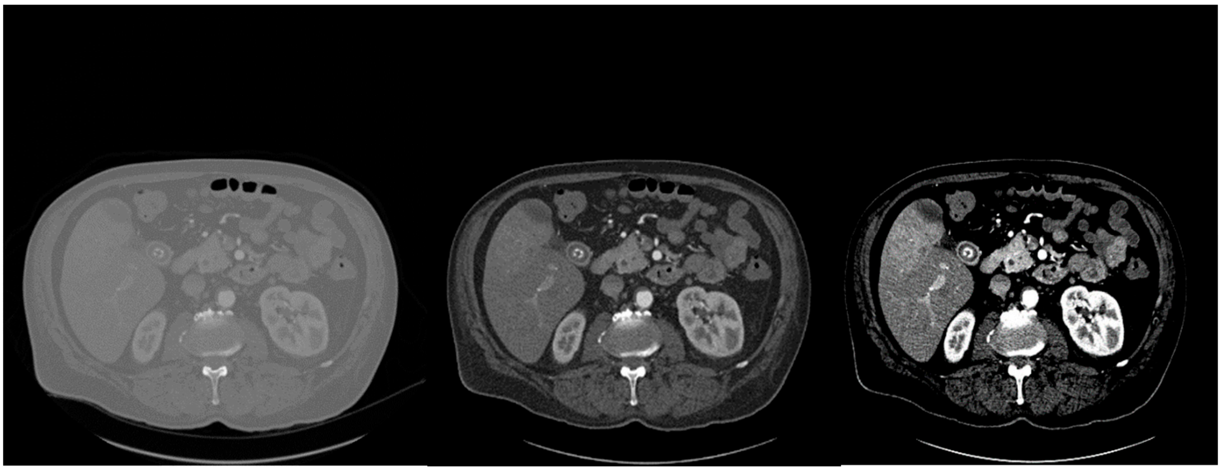

(a) (b) (c)

Figure S1. 2D Kidney CT slices of varying HU. **(a)** Total HU range, original slice, **(b)** range between [-200, 500], as used in the current study, and **(c)** [-30, 200].

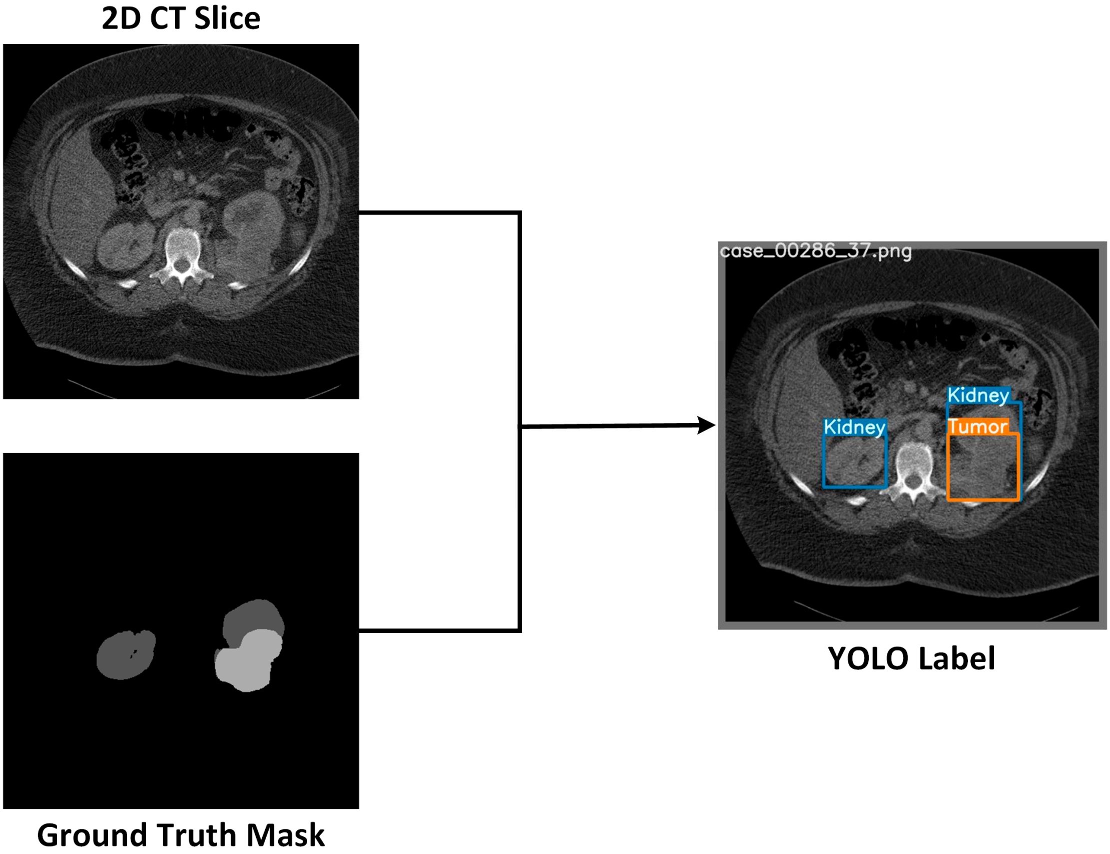

Figure S2. Bounding box creation process for training YOLO frameworks.

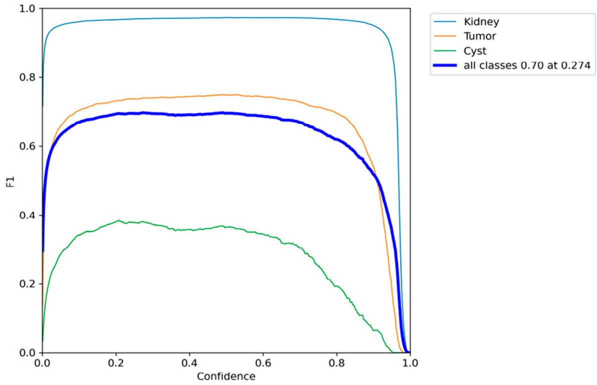

(a) F1-curve

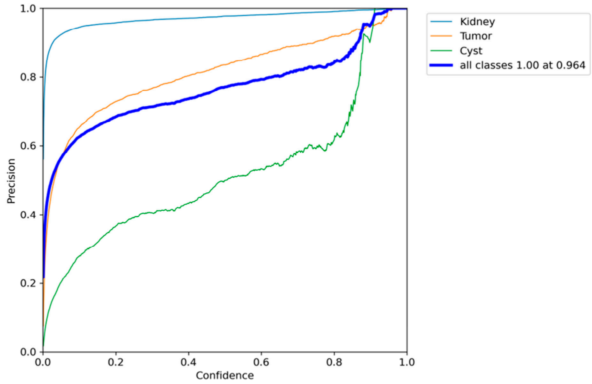

(b) P-curve

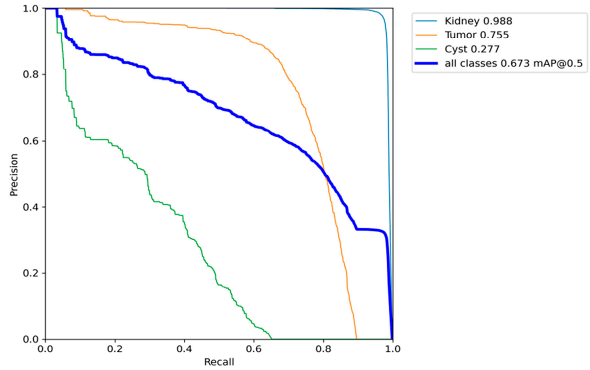

(c) PR-curve

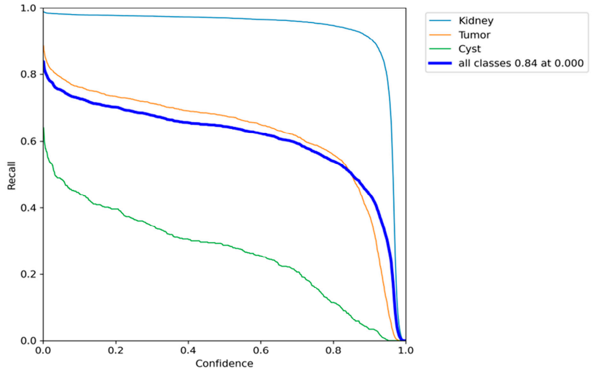

(d) R-curve

Figure S3. YOLOv7 model performance curves plotted for each object type (kidney, tumor, and cyst), alongside combined performance.

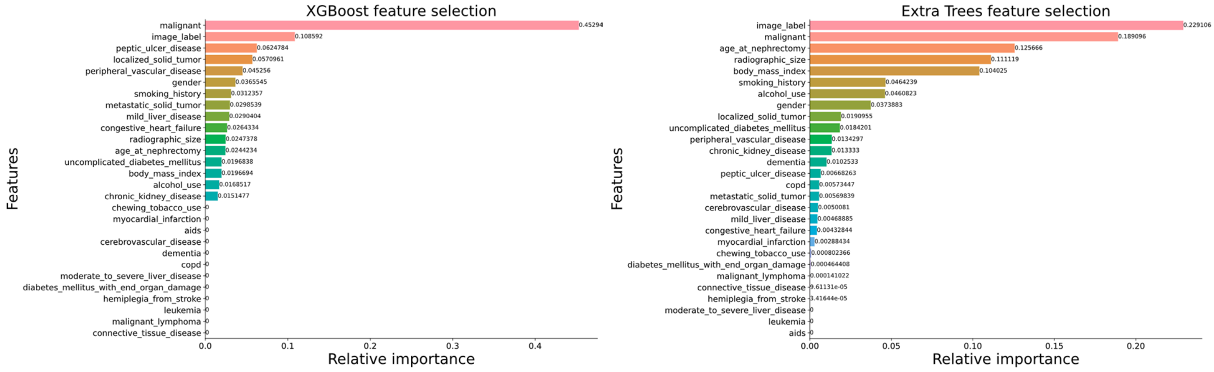

(a) **(b)**

Figure S4. XGBoost **(a)** and Extra Trees **(b)** classifier-based feature selection outcomes for the kidney tumor classification. Ranked features from the Extra trees and Random Forest algorithms were very similar.

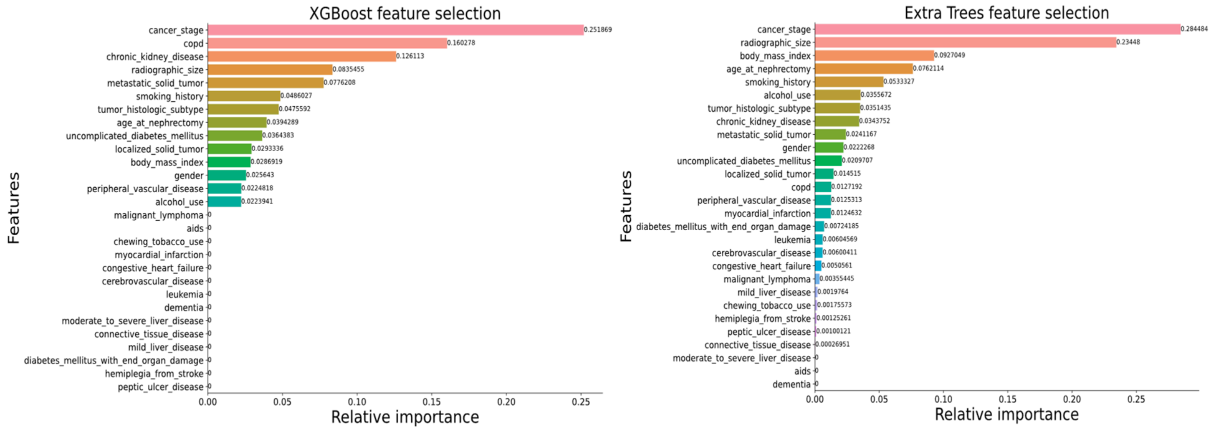

(a) (b)

Figure S5. XGBoost **(a)** and Extra Trees **(b)** classifier-based feature selection outcomes for the surgery procedure classification. Ranked features from the Extra trees and Random Forest algorithms were very similar.

Table S1. Subject-wise split five-fold information regarding the subject range

Fold Num	Train (Sub Range)	Validation (Sub Range)	Test (Sub Range)
**1**	"1-192"	"193-240"	"241-300"
**2**	"(1-132)+(241-300)"	"133-180"	"181-240"
**3**	"(1-72)+(181-300)"	"73-120"	"121-180"
**4**	"(1-12)+(121-300)"	"13-60"	"61-120"
**5**	"109-300"	"61-108"	"1-60"
